# Screening and Identification of Hub Genes in the Corticosteroid Resistance Network in Human Airway Epithelial Cells *via* Microarray Analysis

**DOI:** 10.3389/fphar.2021.672065

**Published:** 2021-10-11

**Authors:** Guangsheng Pei, Nan Ma, Fugang Chen, Liyan Guo, Jing Bai, Jingmin Deng, Zhiyi He

**Affiliations:** ^1^ Department of Respiratory and Critical Care Medicine, The First Affiliated Hospital of Guangxi Medical University, Nanning, China; ^2^ Department of Respiratory and Critical Care Medicine, The First Affiliated Hospital of Henan University of Science and Technology, Luoyang, China

**Keywords:** microarray analysis, bioinformatics analysis, corticosteroid resistance, hub genes, human airway epithelial cells, scopoletin

## Abstract

**Background and Objective:** Corticosteroid resistance is a major barrier to chronic obstructive pulmonary disease (COPD), but the exact mechanism of corticosteroid resistance in COPD has been less well studied.

**Methods:** The microarray dataset GSE11906, which includes genomic and clinical data on COPD, was downloaded from the Gene Expression Omnibus (GEO) database, and the differentially expressed genes (DEGs) were identified using R software. Gene set enrichment analysis (GSEA) and Kyoto Encyclopedia of Genes (KEGG) were utilized to enrich and analyze the gene cohort related to the response to steroid hormones, respectively. The Connectivity Map (CMap) database was used to screen corticosteroid resistance-related drugs that might exert a potential therapeutic effect. STRING was used to construct a protein-protein interaction (PPI) network of the gene cohort, and the CytoHubba plug-in of Cytoscape was used to screen the hub genes in the PPI network. The expression levels of hub genes in cigarette smoke extract (CSE)-stimulated bronchial epithelial cells were assayed by quantitative real-time PCR and western blotting.

**Results:** Twenty-one genes were found to be correlated with the response to steroid hormones. In the CMap database, 32 small-molecule compounds that might exert a therapeutic effect on corticosteroid resistance in COPD were identified. Nine hub genes were extracted from the PPI network. The expression levels of the BMP4, FOS, FN1, EGFR, and SPP1 proteins were consistent with the microarray data obtained from molecular biology experiments. Scopoletin significantly restrained the increases in the levels of AKR1C3, ALDH3A1, FN1 and reversed the decreases of phosphorylated GR and HDAC2 caused by CSE exposure.

**Conclusion:** The *BMP4*, *FOS*, *FN1*, *EGFR*, and *SPP1* genes are closely correlated with CSE-induced glucocorticoid resistance in airway epithelial cells. Scopoletin may be a potential drug for the treatment of glucocorticoid resistance caused by CSE.

## Introduction

Chronic obstructive pulmonary disease (COPD) is estimated to become the third leading cause of death worldwide by 2020. Environmental exposure can cause COPD, and the primary factor is cigarette smoking (López-Campos et al., 2016). Chronic inflammation in the airways and lung parenchyma is a characteristic of COPD, and various inflammatory cytokines are upregulated in the lungs (López-Campos et al., 2016; Barnes, 2019). Although glucocorticoids are the front-line anti-inflammatory therapies for chronic inflammation, most patients with COPD have a poor response (Rossios et al., 2012). The molecular mechanisms of steroid resistance are due to the decreased expression of the glucocorticoid receptor (GR), which is the receptor for corticosteroids (Barnes, 2017). Several molecular pathways are involved in steroid resistance and lead to the downregulation of *GR*, and these pathways include the *PIK3CD/Akt, AP-1*, *P38 MAPK*, and *JNK* pathways (Barnes, 2010), whose activity and expression are upregulated in the lungs of smokers or patients with COPD (Kirkham and Barnes, 2013; Barnes et al., 2015; Fischer et al., 2015). Histone deacetylase-2 (*HDAC2*) can be recruited by corticosteroids, which results in the effective repression of activated inflammatory genes. Cigarette smoking markedly suppresses the activity and expression of *HDAC2* induced by oxidative/nitrative stress and results in inflammation showing resistance to glucocorticoids (Barnes, 2009; Li et al., 2012). However, the effects of the current treatments for corticosteroid resistance are poor. Therefore, finding new targets for treating corticosteroid resistance has potential clinical value.

With the recent advancement of gene chips, genome-wide expression profiles can be obtained using microarray platforms and analyzed using R language and unbiased bioinformatic tools to identify novel hub genes (Sun et al., 2019). Exporting microarray data to modern pathway profiling software could reveal novel targets for treatment (Wei et al., 2019; Xie et al., 2019). Gene set enrichment analysis (GSEA) can help analyze and interpret microarray platform data (Powers et al., 2018). The power of GSEA, which summarizes microarray data of gene expression changes into gene sets, provides a list of genes that are significantly enriched based on their correlation with biological functions and biological processes. This method can enrich genes that are significantly associated with a pathway and phenotype by enabling the detection of gene sets, and its practicality for many applications has been demonstrated (Subramanian et al., 2007; Powers et al., 2018). This method has been successfully used to identify key pathways involved in pancreatic carcinoma (Zhou et al., 2018), reveal seven pathways associated with methyladenosine-related genes in hepatocellular carcinoma (Zhou et al., 2019), and elucidate the role of the TGF-β signaling pathway in preeclampsia (Wu et al., 2019). These results show the potential use of GSEA for studying COPD-related genes and extracting genes involved in corticosteroid resistance from the whole genome.

Here, we applied a bioinformatics method to reveal novel genes associated with corticosteroid resistance in patients with COPD. The “limma” package was used to identify the differentially expressed genes (DEGs), and a GSEA was performed to reveal corticosteroid resistance-related genes. A protein-protein interaction (PPI) network was established using String (https://string-db.org/), and the hub genes were extracted using the Cytoscape software. Furthermore, the corticosteroid resistance-related genes were verified by quantitative real-time polymerase chain reaction (qPCR) and western blot (WB) analyses.

## Materials and Methods

### Microarray Data Collection

The raw microarray dataset of COPD (GSE11906), which was obtained using the Affymetrix human genome U133 plus 2.0 array, was downloaded from the Gene Expression Omnibus (GEO) database (https://www.ncbi.nlm.nih.gov/geo/). The tissue source of these samples was the bronchial airway epithelium. The mRNA expression data were normalized by log2 transformation to obtain the COPD/normal ratio in the GSE11906 dataset.

### GSEA

The “limma” package of R software (version 3.5.1) was used to screen GSE11906 to identify the DEGs, and mRNAs with a *P*-value < 0.05 were considered to be genes with significant differential expression. For the identification of genes associated with the response to steroid hormones, the DEGs were analyzed by a GSEA using Gene Ontology (GO) gene sets accessed from MSigDB. Java GSEA software was used for GSEA, the gene sets from COPD vs normal samples were compared, and the gene sets with an FDR <0.1 and a nominal *p* < 0.05 were regarded as significantly enriched. The distinct expression profiles of genes associated with the response to steroid hormones are shown as a heatmap.

### Functional Enrichment Analysis

To further research the functional implications of the DEGs associated with the response to steroid hormones, we performed Kyoto Encyclopedia of Genes and Genomes (KEGG) functional enrichment analyses. The results from the KEGG enrichment analyses were based on the criterion *P*-value < 0.05. The significant enrichment results were visualized using R software.

### PPI of Corticosteroid Resistance-Related Genes and Identification of Hub Genes

After screening for genes associated with the response to steroid hormones based on module connectivity, the genes that were found to be enriched using the GSEA software were uploaded to the Search Tool for the Retrieval of Interacting Genes (STRING) database to study the interactions among these genes, as well as *NR3C1/GR* and *HDAC2*. The interaction data for the above-mentioned genes were downloaded from STRING and analyzed using Cytoscape (version: 3.5.1) to construct a PPI network. Furthermore, Cytoscape software was used to analyze the PPI network to identify the hub genes (Yi et al., 2018; Zhou et al., 2018). CytoHubba in Cytoscape was used to select the hub genes in the PPIs, and the analysis was performed using the default parameters.

### Screening of Potential Drugs for Corticosteroid Resistance Treatment Based on a Connectivity Map

The CMap database was used to screen small molecules with therapeutic potential in corticosteroid resistance. The DEGs associated with corticosteroid resistance were uploaded to the CMap database and analyzed, and a negative connectivity score suggested that the drug might reverse the signature biology of human cells and show its potential therapeutic value (Liu et al., 2020). The small molecules were identified based on the following criteria: *P*-value < 0.05 and a negative connectivity score.

### Validation of Key Genes Through Biological Experiments

#### BEAS-2B Cell Culture and Treatment

The human bronchial epithelial cell line BEAS-2B (ATCC® CRL-9609™, Shanghai Cell Bank, Chinese Academy of Sciences) was maintained in RPMI 1640 medium (Gibco, New York, NY, United States) supplemented with 10% fetal bovine serum (Gibco) at 37°C under 5% CO_2_ conditions. BEAS-2B cells were treated with cigarette smoke extract (CSE) for 24 h and collected for the extraction of RNA and proteins for qPCR and WB assays, respectively.

### CSE Preparation

CSE was prepared according to the protocol from our previous study (Li et al., 2012). The smoke of 10 unfiltered cigarettes was slowly dissolved in 20 ml of RPMI-1640 medium. The pH of the CSE solution was adjusted to 7.4, and the CSE solution was filtered through a 0.22-μm leach to remove bacteria. The optical density was measured at a wavelength of 320 nm. The CSE solution was used in the experiments within 1 h of extraction. A CSE concentration of 0.1% was used for the study based on the results obtained using the Cell Counting Kit-8 (CCK-8) assay.

### Validation of the Results by qPCR and WB Analyses

Total RNA from the CSE-treated and control cells was extracted using the TRIzol reagent (Invitrogen, Carlsbad, CA, United States). The primer sequences are listed in Table 1, and the total RNA quality and quantity were analyzed with a spectrophotometer (Thermo, United States). RNA was reverse transcribed into cDNA using the PrimeScript^TM^ RT reagent kit with gDNA Eraser (TaKaRa, Dalian, China), and mRNA was prepared using kits. qPCR was performed using SYBR® Premix Ex Taq^TM^ II (Takara, Dalian, China). The procedures were performed according to the manufacturers’ instructions. The protein concentration in the lysate of BEAS-2B cells stimulated with 0.1% CSE for 24 h was determined by the bicinchoninic acid method. Twenty micrograms of protein was electrophoresed on a sodium dodecyl sulfate polyacrylamide gel and then electroblotted onto polyvinylidene difluoride membranes. After blocking with 5% skim milk, the membranes were incubated with the corresponding primary antibodies overnight in a shaker at 4°C. The following primary antibodies were used in this study: anti-BMP4 (1:1000; A11405, ABclonal), SPP1 (1:1000; A11405, ABclonal), ALDH3A1 (1:1000; A13275, ABclonal), AKR1C3 (1:1000; A1781, ABclonal), c-Fos (1:1000; #2250, Cell Signaling Technology), FN1 (1:1000; #26836, Cell Signaling Technology), EGFR (1:1000; #4267, Cell Signaling Technology), GR (1:1000; #12041, Cell Signaling Technology), phospho (P)-GR (1:1000; #4161, Cell Signaling Technology), HDAC2 (1:1000; #2540, Cell Signaling Technology), and GAPDH (1:4,000; AF7021, Affinity). The expression of proteins was detected using the Odyssey system after incubation with a secondary antibody (1:20,000; #5151, Cell Signaling Technology). Scopoletin (S2500, Sigma-Aldrich) was considered a compound that could potentially alleviate steroid resistance according to the results of CMap database analysis. WB analysis was used to confirm the influence of scopoletin on the hub genes.

**TABLE 1:** Primers used for qPCR in this study.

Gene	Primer sequence (5′–3′)
FN1-Forward	AAA​TGG​CCA​GAT​GAT​GAG​C
FN1-Reverse	TAA​CAC​GTT​GCC​TCA​TGA​G
EGFR-Forward	ATT​TAC​AGG​AAA​TCC​TGC​ATG​G
EGFR-Reverse	TCA​CTG​CTG​ACT​ATG​TCC​C
CCL2-Forward	AGA​AGC​TGT​GAT​CTT​CAA​GAC
CCL2-Reverse	GTC​CAT​GGA​ATC​CTG​AAC​C
IL1B-Forward	GCT​TAT​TAC​AGT​GGC​AAT​GAG​G
IL1B-Reverse	AGA​TTC​GTA​GCT​GGA​TGC​C
C3-Forward	GTG​CAA​AGA​GGA​CTG​TGA​G
C3-Reverse	ATG​AAG​CAA​TTC​TCC​TCA​GC
SPP1-Forward	TGC​CGT​GAT​TCA​GTA​CCC​TG
SPP1-Reverse	AAC​GGG​GAT​GGC​CTT​GTA​TG
NQO1-Forward	CTC​CTA​TGA​ACA​CTC​GCT​C
NQO1-Reverse	ACC​TTG​TGA​TAT​TCC​AGT​TCC
FOS-Forward	TGG​GCT​TCC​CAG​AAG​AGA​TG
FOS-Reverse	TGA​GGA​GAG​GCA​GGG​TGA​A
BMP4-Forward	AGC​ACT​GGT​CTT​GAG​TAT​CC
BMP4-Reverse	CTC​CAG​ATG​TTC​TTC​GTG​GT
ALDH3A1-Forward	TGT​TCT​CCA​GCA​ACG​ACA​AGG
ALDH3A1-Reverse	AGG​GCA​GAG​AGT​GCA​AGG​T
AKR1C3-Forward	GTC​ATC​CGT​ATT​TCA​ACC​GGA​G
AKR1C3-Reverse	CCA​CCC​ATC​GTT​TGT​CTC​GTT
GAPDH-Forward	TGG​GCT​TCC​CAG​AAG​AGA​TG
GAPDH-Reverse	TGG​TGA​AGA​CGC​CAG​TGG​A

### Statistical Analysis

The qPCR and WB data are presented as the means ±SDs. Three repeated independent experiments were performed from each group. Statistical comparisons were performed using the SPSS 18.0 software (Chicago, IL, United States). A two-tailed Student’s *t*-test was used for comparison between two groups. One-way analysis of variance was conducted to evaluate significant differences among multiple groups. The results with *p* < 0.05 were considered statistically significant.

## Results

### Identification of Differentially Expressed Genes

**FIGURE 1:**
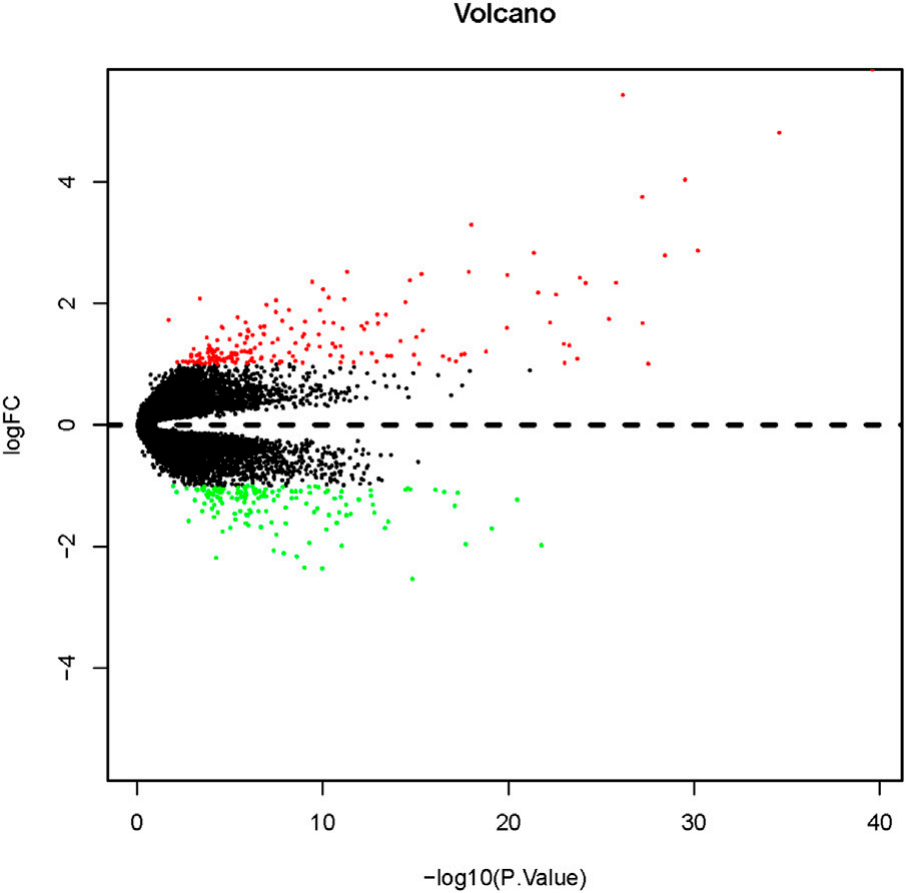
Volcano plot of DEG expression in the microarray. The red points in the volcano plot are the mRNAs showing significantly higher expression, and the green points show the mRNAs with significantly lower expression. The differences are set as a fold change ≥1.5 and a P-value ≤ 0.05. FC, fold change.

Thirty-three COPD samples from long-term smokers and 72 normal samples from nonsmokers were included in this study. The main clinical features of the participants are listed in Table 2. A total of 335 DEGs, including 187 upregulated and 148 downregulated genes, were obtained from GSE11906 using the limma package. Volcano plots of the DEGs are shown in Figure 1 (fold change ≥1.5 and *P*-value ≤ 0.05).

**TABLE 2:** Clinical information of the study participants. GOLD: The Global Initiative for Chronic Obstructive Lung Disease.

Variables	Normal: 72	COPD: 33
Sex	Males: 53	Males: 26
	Females: 19	Females: 7
Age	≤40: 36	≤40: 1
	>40: 36	>40: 32
Smoking Status	None	Long-term
Pack-Years	—	≤10: 0
		>10: 33
GOLD stages	—	I: 9
		II: 9
		III: 2
		Early: 13
Ethnicity	Asian: 2	Asian: 1
	Black: 30	Black: 15
	White: 39	White: 17
	Black/Hispanic: 1	Black/Hispanic: 0
Sample source	Large airways: 20	Large airways: 0
	Small airways: 35	Small airways: 33
	Trachea: 17	Trachea: 0

### GSEA of DEGs

**FIGURE 2:**
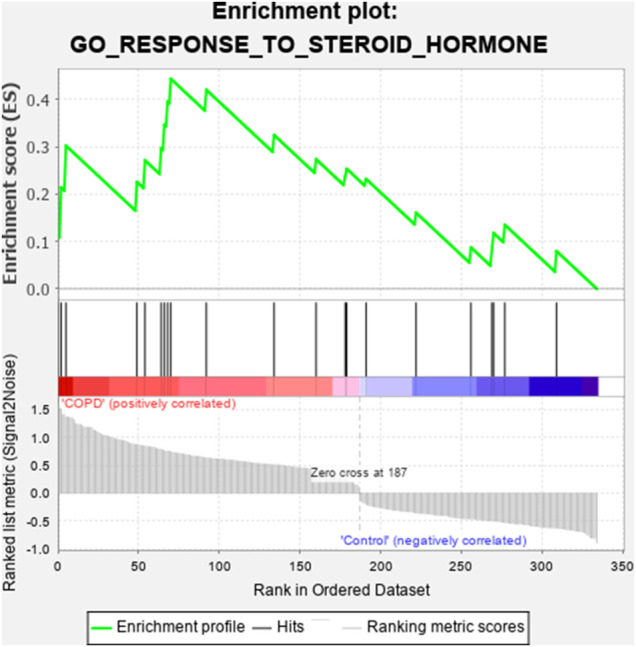
GSEA plot of steroid hormone-related genes in cigarette smoke-induced COPD.

**FIGURE 3:**
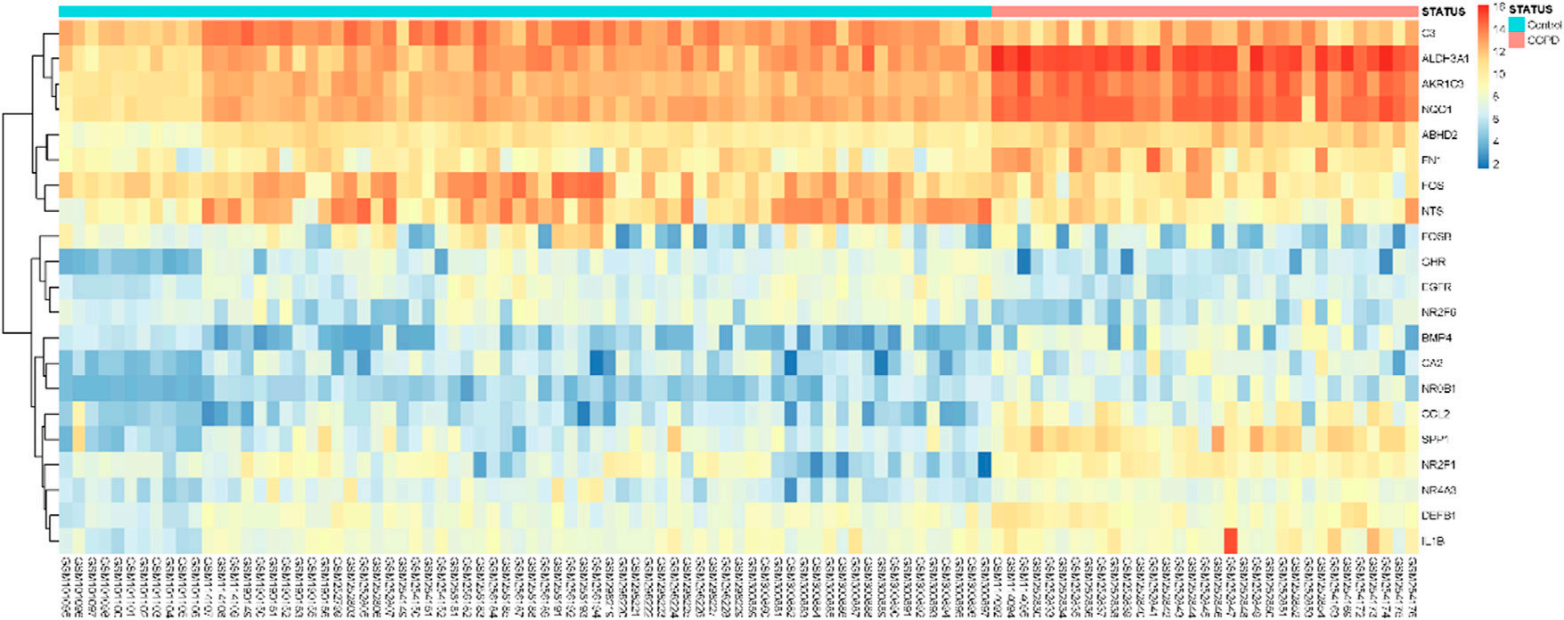
Heatmap of DEGs related to the response to steroid hormones. The blue coded bar above the heat map represents the normal samples and the red coded bar represents the copd samples. Red dots represent upregulated DEGs, and blue dots represent downregulated DEGs.

GSEA of the DEGs was conducted using GSEA software based on the following filter conditions: *P*-value < 0.05 and FDR *q*-value < 0.05. The GSEA plots of the genes enriched in the response to steroid hormones are shown in Figure 2. The 21 genes related to the response to steroid hormones that were found to be enriched by the GSEA are shown in Table 3, and the expression of these 21 genes is shown in the heatmap diagram presented in Figure 3.

**TABLE 3:** Twenty-one genes related to the response to steroid hormones determined by GSEA.

Probe	Rank metric score	Running ES	Core enrichment
NQ O 1	1.617913	0.112354	Yes
ALDH3A1	1.507612	0.213863	Yes
AKR1C3	1.364684	0.302262	Yes
NR2F1	0.868267	0.225615	Yes
ABHD2	0.840391	0.271236	Yes
NR0B1	0.779968	0.296737	Yes
SPP1	0.762481	0.346502	Yes
BMP4	0.74651	0.395158	Yes
CCL2	0.741474	0.443463	Yes
DEFB1	0.627186	0.420138	No
FN1	0.505603	0.324676	No
CA2	0.411556	0.273638	No
NR4A3	0.253706	0.237116	No
IL1B	0.233349	0.253321	No
NR2F6	−0.19669	0.231948	No
FOSB	−0.34914	0.160652	No
FOS	−0.45696	0.08729	No
EGFR	−0.49457	0.083418	No
GHR	−0.49563	0.117836	No
C3	−0.52379	0.135101	No
NTS	−0.6227	0.079618	No

### KEGG Enrichment Analysis

**FIGURE 4:**
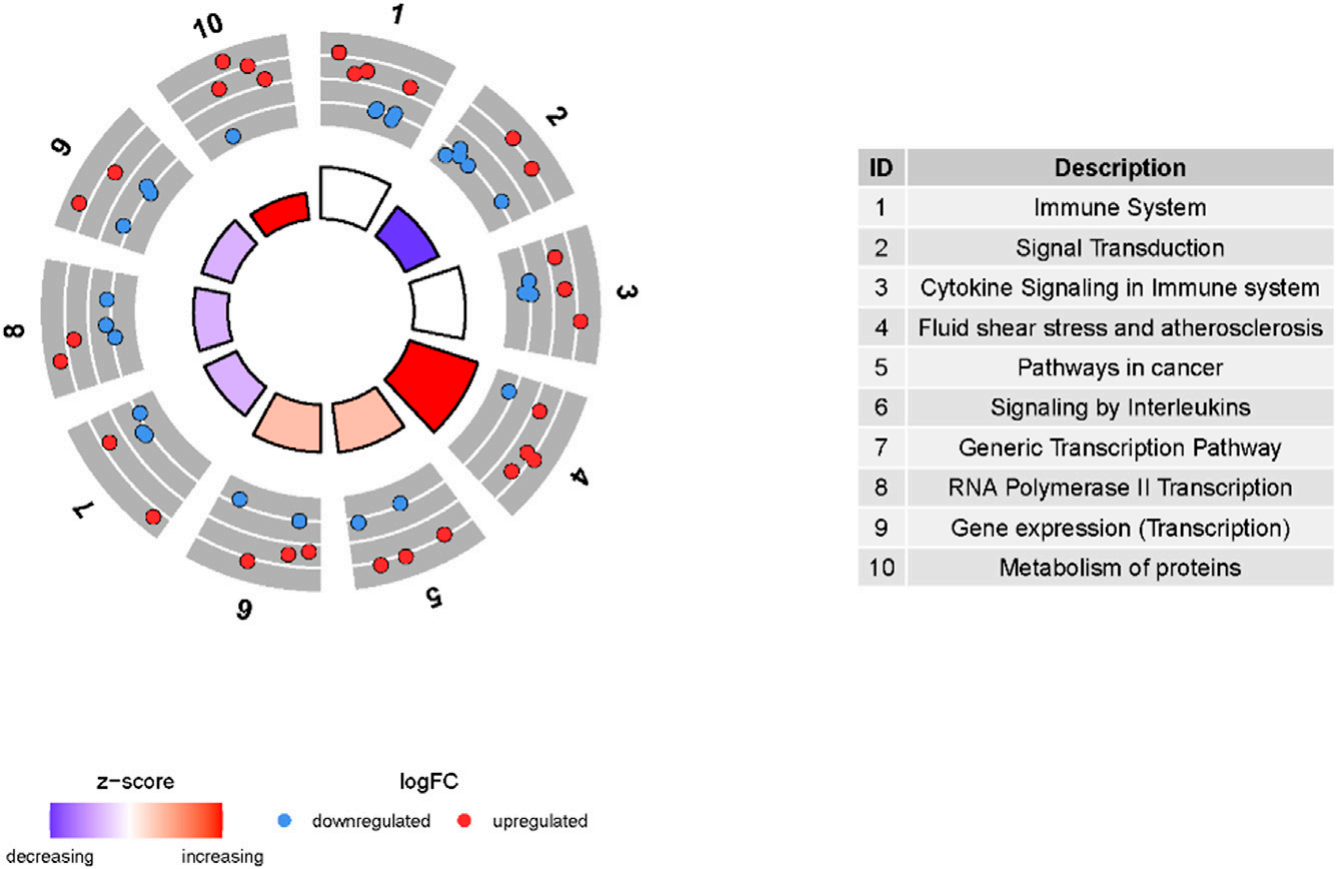
KEGG enrichment analysis of the 21 genes related to the response to steroid hormones. The outer circle represents the number of genes enriched in the corresponding pathway. Red dots indicate upregulated DEGs and blue dots indicate downregulated DEGs. The inner circle represents the Z-score. Red indicates more enrichment of upregulated DEGs, and purple represents more enrichment of downregulated DEGs.

To study the functional implications of the 21 above-mentioned genes, we performed a KEGG functional enrichment analysis. The results showed that these genes were significantly enriched in the immune system, signal transduction, cytokine signaling in the immune system, fluid shear stress, atherosclerosis and pathways in cancer (Figure 4).

### Construction of the PPI Network and Identification of Hub Genes

**FIGURE 5:**
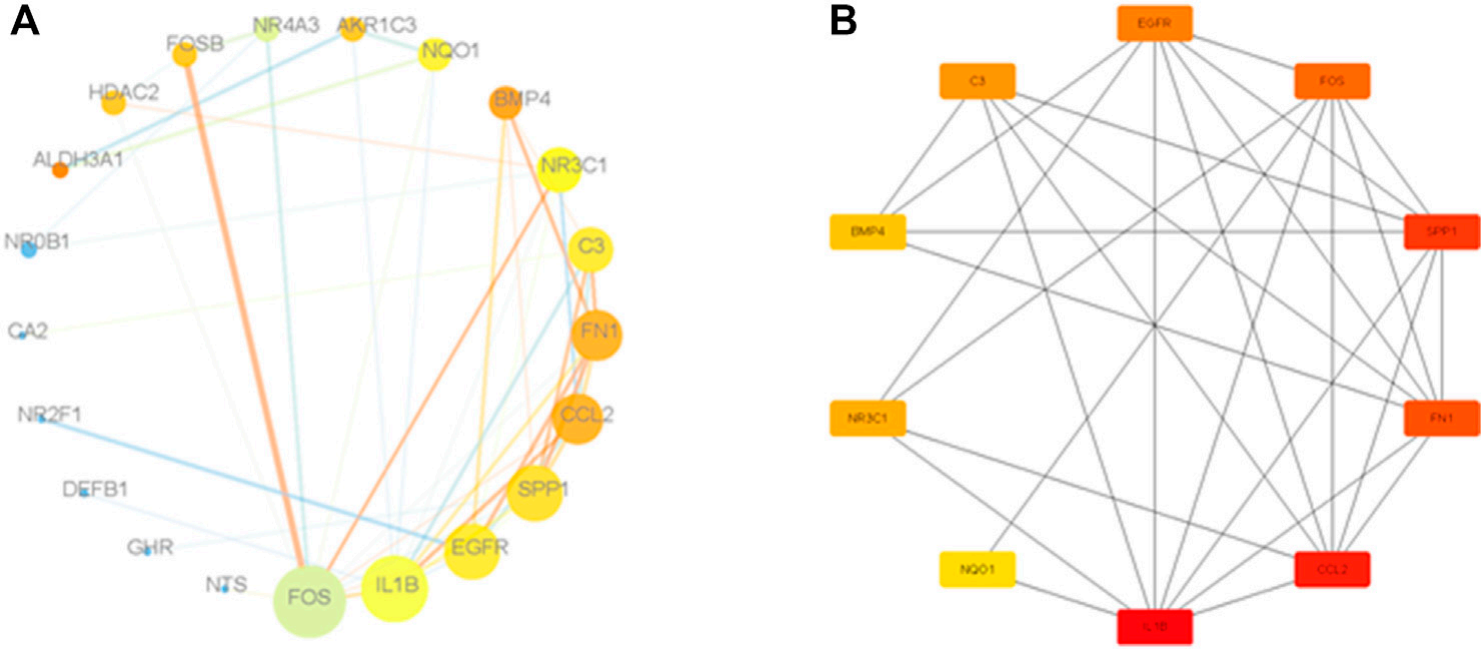
PPI network of genes associated with glucocorticoid resistance. **(A)**: PPI network of 21 genes associated with glucocorticoid resistance. The node size indicates the level of the gene in the PPI network, and the node color depth indicates the level of the clustering coefficient. The thickness of the edge lines indicates the size of the coexpression relationship between the genes, and the color depth of the edge lines indicates the levels of the interaction coefficients between the genes. **(B)**: PPI of nine hub genes in the 21-gene cohort associated with glucocorticoid resistance. A darker node color indicates that the gene plays a more critical role in the network.

The 21 above-mentioned genes, as well as *NR3C1* and *HDAC2*, were uploaded to STRING to study their interactions, and PPIs were plotted using Cytoscape based on the interaction data obtained from STRING (Figure 5A). In the PPI network of the 21 genes related to the response to steroid hormones in COPD and *NR3C1*, the top nine hub genes with the highest criticality were *EGFR*, *FOS*, *SPP1*, *FN1*, *CCL2*, *IL1B*, *NQ O 1*, *BMP4*, and *C3* (Figure 5B). Therefore, we speculated that these nine hub genes might play an important role in corticosteroid resistance in smoke-induced COPD.

### Verification of Hub Genes

**FIGURE 6:**
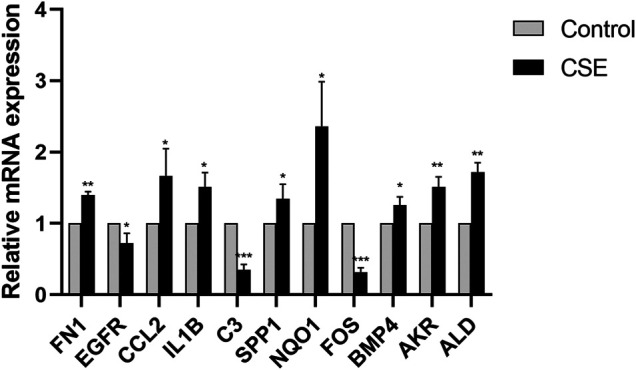
Relative mRNA expression levels of the hub genes in 0.1% CSE-stimulated Beas-2B cells (*p < 0.05, **p < 0.01, ***p < 0.001).

**FIGURE 7:**
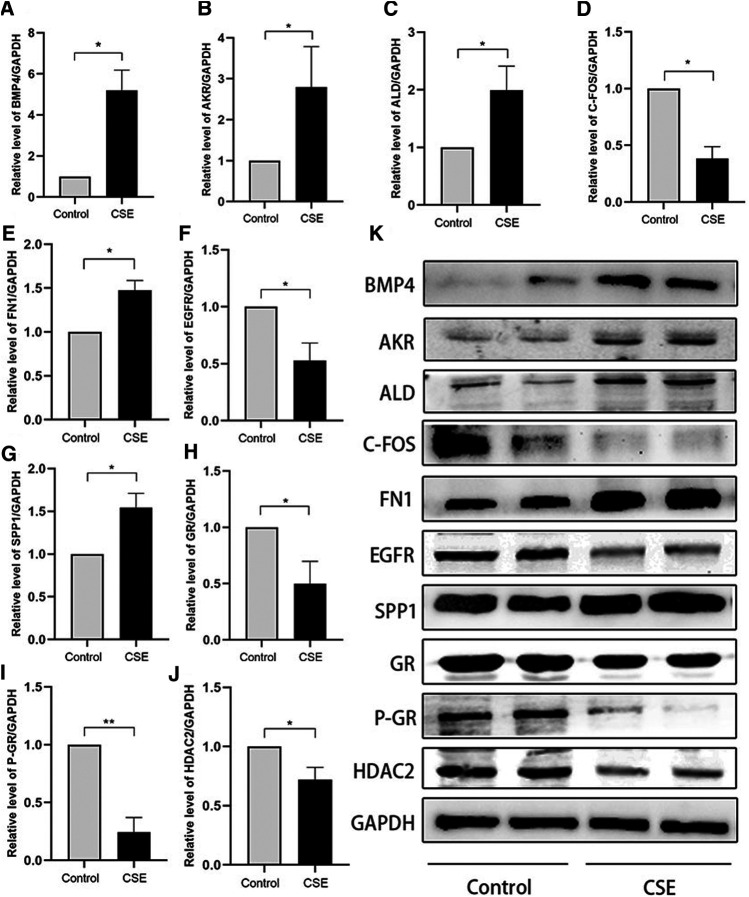
Relative protein expression levels determined by western blot analysis. **(A)** BMP4, **(B)** AKR1C3, **(C)** ALDH3A1, **(D)** C-FOS, **(E)** FN1, **(F)** EGFR, **(G)** SPP1, **(H)** GR, **(I)** P-GR and **(J)** HDAC2 (*p < 0.05, **p < 0.01).

To verify the hub genes extracted from the PPI network, we determined their mRNA and protein expression levels in CSE-treated human bronchial epithelial cells using qPCR and western blot analyses. In addition, we verified the expression of *ALDH3A1* and *AKR1C3*, which were significantly upregulated in patients with COPD and belonged to the core enrichment genes according to the microarray analysis. The qPCR results indicated that the expression of all hub genes, as well as *ALDH3A1* and *AKR1C3*, was consistent with the data from the selected microarray datasets (Figure 6). The WB data showed that the expression levels of the C-FOS, EGFR, GR, P-GR, and HDAC2 proteins were significantly downregulated in the CSE group. The expression levels of the BMP4, FN1, SPP1, ALDH3A1, and AKR1C3 proteins in BEAS-2B cells increased after stimulation with 0.1% CSE (*p* < 0.05; Figure 7). No significant differences in the expression of the CCL2, IL1B, C3, and NQO1 proteins were found between the CSE and control groups.

### Prediction and Verification of Potential Agents for the Treatment of CSE-Induced Corticosteroid Resistance

**FIGURE 8:**
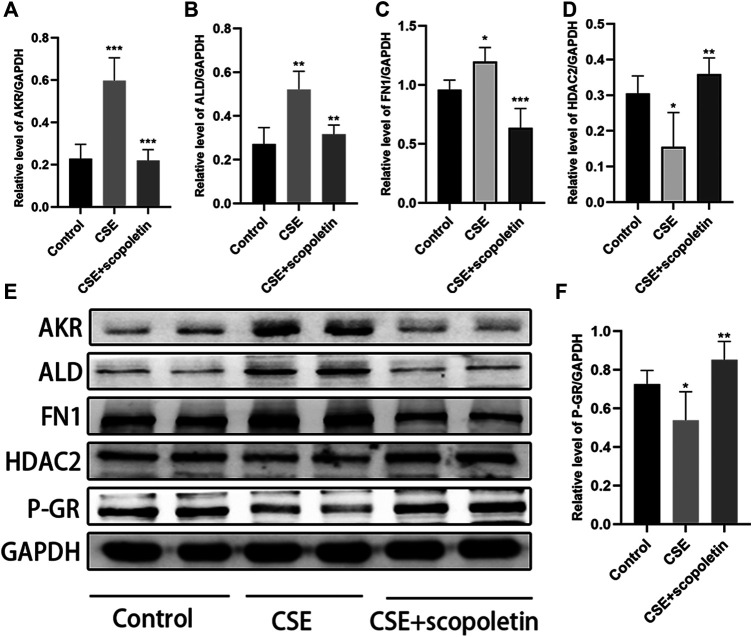
Scopoletin contributes to the recovery of the indicators of CSE-induced glucocorticoid resistance. **(A)** AKR1C3, **(B)** ALDH3A1, **(C)** FN1, **(D)** HDAC2, **(F)** P-GR (*p < 0.05, **p < 0.01, ***p < 0.001).

Small-molecule compounds that might alleviate corticosteroid resistance were predicted through CMap analysis. The results suggest that 32 compounds might exert a therapeutic effect on corticosteroid resistance induced by oxidative stress in COPD (Table 4). The most promising small-molecule compounds are scopoletin, harmol, AG-012559, AH-23848, perhexiline, and fluorometholone. We chose scopoletin as a potential agent for its ability to inhibit the activation of nuclear factor-κB (NF-κB) against COPD-associated oxidative stress (Leema and Tamizhselvi, 2018). A scopoletin concentration of 100 μM was chosen as the final intervention concentration according to the results of our CCK-8 assay, and the compound was added to the medium 2 h before CSE treatment. The results indicated that scopoletin significantly restrained the increases in the levels of AKR1C3, ALDH3A1 and FN1 caused by CSE exposure. In addition, scopoletin obviously reversed the decreases in P-GR and HDAC2 levels (Figure 8).

**TABLE 4:** Small-molecule compounds identified as potential drugs for corticosteroid resistance treatment by CMap analysis.

CMap name	Mean	*n*	Enrichment	*p*	Specificity	Percent non-null
scopoletin	−0.543	2	−0.857	0.04066	0.0385	100
harmol	−0.602	4	−0.837	0.00123	0.0273	100
AG-012559	−0.583	3	−0.835	0.00895	0.0109	100
AH-23848	−0.527	3	−0.781	0.02149	0	100
perhexiline	−0.524	4	−0.779	0.00491	0.0252	100
fluorometholone	−0.506	4	−0.759	0.00688	0.019	100
Penbutolol	−0.234	3	−0.746	0.03327	0.0444	66
harmalol	−0.431	3	−0.733	0.03936	0.0462	66
trapidil	−0.417	3	−0.73	0.04052	0.0926	66
PF-01378883-00	−0.388	4	−0.712	0.01387	0.0058	75
glycocholic acid	−0.288	4	−0.703	0.01605	0.0058	75
metitepine	−0.492	4	−0.7	0.01701	0.0174	75
kaempferol	−0.535	4	−0.698	0.01772	0.0333	75
maprotiline	−0.349	4	−0.697	0.01774	0.017	75
domperidone	−0.407	4	−0.694	0.01866	0	75
fluticasone	−0.315	4	−0.687	0.02085	0.1083	75
abamectin	−0.329	4	−0.671	0.0262	0.0432	75
methyldopate	−0.236	4	−0.67	0.0265	0.073	50
ciclacillin	−0.272	4	−0.664	0.02871	0.0223	50
bambuterol	−0.353	4	−0.66	0.03018	0.0186	75
sulfametoxydiazine	−0.343	4	−0.658	0.03127	0.0777	75
SB-202190	−0.259	5	−0.637	0.01632	0.0099	60
N6-methyladenosine	−0.454	4	−0.634	0.04343	0.0828	75
biperiden	−0.29	5	−0.633	0.01712	0.2381	80
halcinonide	−0.413	5	−0.575	0.04175	0.0506	80
benzamil	−0.256	6	−0.559	0.02751	0.0387	50
PNU-0251126	−0.316	6	−0.535	0.0402	0.1133	66
diphenylpyraline	−0.366	6	−0.532	0.04195	0.1375	66
wortmannin	−0.412	18	−0.496	0.0002	0.1147	66
fludrocortisone	−0.305	8	−0.472	0.03757	0.3732	62
thioridazine	−0.288	20	−0.391	0.00294	0.2473	60
LY-294002	−0.336	61	−0.373	0	0.2209	55

## Discussion

The imbalance of pulmonary oxidants and antioxidants caused by long-term exposure to cigarette smoke is thought to be the main driving mechanism of COPD (Kirkham and Barnes, 2013). Several studies have demonstrated that oxidative stress and inflammation in COPD are not only local but also interdependent and closely related processes in systemic inflammation in the lungs (Sundar et al., 2013). Cigarette smoke-mediated oxidative stress and inflammation induce a number of kinase signaling pathways that are considered to be associated with steroid resistance (Liao et al., 2020). Bronchial/lung epithelial cells are the first barrier against inhaled cigarette smoke. Oxidative stress induced by cigarette smoke promotes the release of various cytokines and chemokines in epithelial cells. Epithelial damage and repair play a central role in the pathogenesis of COPD (Heijink et al., 2014). Previous studies have shown that the stimulation of lung epithelial cells with cigarette smoke can result in corticosteroid insensitivity. An important molecular mechanism leading to glucocorticoid resistance has been shown to be a result of decreases in the expression and activity of GR and HDAC2 caused by cigarette smoke exposure (Barnes, 2017).

Our study extracted DEGs from the GEO database of COPD at the whole gene level, and 21 enriched genes correlated with the response to steroid hormones were identified from a GSEA of these DEGs. This gene cohort included *NQ O 1*, *ALDH3A1*, *AKR1C3*, *NR2F1*, *ABHD2*, *NR0B1*, *SPP1*, *BMP4*, *CCL2*, *DEFB1*, *FN1*, *CA2*, *NR4A3*, *IL1B*, *NR2F6*, *FOSB*, *FOS*, *EGFR*, *GHR*, *C3*, and *NTS*. The nine core genes in the gene cohort, which were considered to make a major contribution to the enrichment score of the gene set, were *NQO1*, *ALDH3A1*, *AKR1C3*, *NR2F1*, *ABHD2*, *NR0B1*, *SPP1*, *BMP4*, and *CCL2*. We also analyzed the 21-gene cohort through the KEGG database. The results showed that the genes in the cohort were enriched in the immune system, signal transduction, cytokine signaling in the immune system, fluid shear stress, atherosclerosis, and pathways in cancer. These results indicate that the functions of the 21 genes are closely correlated with the immune response. Furthermore, we constructed a PPI network to assess the relationships among the 21 genes identified in the cohort, as well as *HDAC2* and *GR*. The following hub genes of the 21-gene PPI network were extracted using the Cytoscape plug-in of CytoHubba: *FN1*, *EGFR*, *CCL2*, *IL1B*, *C3*, *SPP1*, *NQO1*, *FOS*, and *BMP4*.

A previous study demonstrated that miR-183 regulates LPS-induced oxidative stress in rat hippocampal neurons by regulating the expression of FN1 to reduce oxidative damage (Xie et al., 2020). Nicotine and cigarette smoke obviously stimulate the expression of FN1 in lung fibroblasts and mouse lung tissues (Roman et al., 2004; Zhao et al., 2019). EGFR plays an important role in cancer progression and regulates inflammation and oxidative stress (Fang et al., 2016). EGFR was shown to be activated by the meprin alpha metalloproteinase to mediate oxidative stress in macrophages (Wang et al., 2017). Additionally, EGFR suppresses the activation of transcription factors, such as NF-κB, and induces proinflammatory gene transcription in macrophages. CCL2 and IL1B are common cytokines involved in chronic inflammation in COPD (Sethi et al., 2012; Barnes, 2016). Cigarette smoke can induce macrophages to release inflammatory mediators, including CXCL1, CXCL8, and CCL2. The increased release of IL1 leads to an increase in the number of macrophages in the lungs of smokers and patients with COPD (Barnes, 2016). Cigarette smoke-induced reactive oxygen species (ROS) can induce IL1B generation by activating Toll-like receptors and inducing immune responses (Zuo et al., 2014). C3 activation is closely related to oxidative stress in mice. Mice with transient focal cerebral ischemia with lower ROS levels show a better neurological outcome, and the inhibition of C3 activation in mice leads to an anti-inflammatory effect (Yang et al., 2013). SPP1 is a molecule with a complex function that decreases the oxidative stress effect and has been implicated in inflammation in the kidney (Trostel et al., 2018). Additionally, SPP1 might exert proinflammatory effects in vascular endothelial cells induced by cigarette smoke and might contribute to inflammation in smokers (Bishop et al., 2012).

NQO1 protein is highly expressed in various cells, such as vascular endothelial cells, epithelial cells, and adipocytes. A number of studies have shown that NQO1 exerts antioxidant effects by reducing the level of ROS (Jo et al., 2016). However, no significant upregulation was observed at the protein level in CSE-treated BEAS-2B cells. FOS is a subunit of AP-1, which is a proinflammatory transcription factor sensitive to oxidative stress that interacts with GR proteins (Frenkel et al., 2015). The overexpression of AP-1 can prevents the binding of GR to glucocorticoid response elements and other proinflammatory DNA-binding transcription factors via PPIs with GR (Pujolsa et al., 2009; Jacques et al., 2010). Previous studies have shown that glucocorticoid resistance in asthma is correlated with the overexpression of AP-1 (Jacques et al., 2010). BMP4 is a new proinflammatory marker that stimulates the production of ROS and induces monocyte adhesion (Tian et al., 2012). BMP4 was found to be upregulated in the airway epithelium of asymptomatic smokers and patients with COPD. The accumulation of BMP4 aggravates airway remodeling by changing the phenotypes of basal stem/progenitor cells (Zuo et al., 2019). AKR1C3 is believed to be closely related to the metabolism of steroids. It can be activated by oxidative stress factors and leads to radiotherapy resistance in lung cancer tissues (Xie et al., 2013). ALDH3A1 was found to be upregulated in lung tissue after exposure to carcinogenic aldehydes (Patel et al., 2008).

To study whether the nine hub genes are altered by CSE stimulation in BEAS-2B cells, we determined the expression of these genes using qPCR and WB assays. Our results demonstrated that the changes in the mRNA expression of the nine genes were consistent with the results from the analysis of the microarray data. The expression levels of the BMP4, FOS, FN1, EGFR, SPP1, ALDH3A1, and AKR1C3 proteins were consistent with the mRNA results. The results demonstrate that CSE could induce glucocorticoid resistance by regulating these genes. The inconsistency between the mRNA and protein levels of CCL2, IL1B, C3, and NQO1 may be related to post-transcriptional modification or post-translational protein processing, specific mechanism needs further study.

CMap is a database covering diseases, gene expression patterns, and interactions between small-molecule compounds (Gao et al., 2019; Wei et al., 2020). This database is an effective genome-based tool for screening new chemopreventive drugs and is commonly used to find small-molecule compounds that exert therapeutic effects on certain diseases (Wei et al., 2020; Yu et al., 2020). We used CMap for drug discovery and identified small-molecule compounds as potential drugs for corticosteroid resistance treatment. Our results showed that 32 small-molecule compounds might exert potential therapeutic effects. Some compounds have been found to exert antioxidative stress effects in other fields. For example, in yeast cells, harmol and harmalol have a significant protective effect against oxidative stress induced by H_2_O_2_ and paraquat. The antigenotoxic and antimutagenic effects of harmol result from its ability to scavenge hydroxyl radicals (Moura et al., 2007). Scopoletin efficiently quenches oxidative stress by stabilizing the nuclear factor erythroid 2-related factor 2 (Nrf2)/antioxidant responsive element (ARE) pathway through enhancement of the nuclear translocation and phosphorylation of Nrf2 (Narasimhan et al., 2019). We chose scopoletin as a candidate compound that might alleviate steroid resistance based on the results of the CMap database analysis. The results of treatment indicated that scopoletin significantly restrained the increases in the AKR1C3, ALDH3A1, and FN1 levels caused by CSE exposure and reversed the decreases in the P-GR and HDAC2 levels. Thus, scopoletin may contribute to the reduction of CSE-induced glucocorticoid resistance in airway epithelial cells. However, the specific molecular regulatory mechanism and how these genes interact to regulate corticosteroid resistance still need to be clarified in future experiments.

In summary, we extracted and validated seven genes that were correlated with corticosteroid resistance and found that they interacted with *GR* and *HDAC2*. However, the study has some limitations. First, we have no direct evidence showing the mutual regulatory relationships among the hub genes, and further studies are needed to validate these relationships. Second, animal model validation is needed for future mechanistic research.

## Conclusion

In this study, we extracted 21 DEGs by integrating the microarray data of COPD and identified a set of enriched genes related to the response to steroid hormones. It was found that the *BMP4*, *FOS*, *FN1*, *EGFR*, and *SPP1* genes were closely correlated with CSE-induced glucocorticoid resistance in airway epithelial cells. In addition, scopoletin was found to be a potential drug for the treatment of glucocorticoid resistance caused by CSE. We hope this research can contribute to the development of corticosteroid resistance therapy for COPD in the future.

## Data Availability

The original contributions presented in the study are included in the article/Supplementary Material, further inquiries can be directed to the corresponding author.
